# Lack of Dietary Diversity Contributes to the Gaps in Micronutrient Status and Physical Development between Urban and Rural Infants

**Published:** 2018-07

**Authors:** Shanshan GENG, Jingqiu MA, Shanshan LIU, Jie ZHANG, Xiaoyang SHENG

**Affiliations:** 1. Dept. of Children and Adolescents Health Care, Xin Hua Hospital Affiliated Shanghai Jiao Tong University School of Medicine, Shanghai, China; 2. MOE and Shanghai Key Lab of Children’s Environmental Health, Shanghai Institute for Pediatric Research, Xin Hua Hospital Affiliated to Shanghai Jiao Tong University School of Medicine, Shanghai, China; 3. Dept. of Anesthesia, Children’s Hospital, School of Medicine, Zhejiang University, Hangzhou, Zhejiang, China

**Keywords:** Dietary diversity, Dietary intake, Micronutrient, Infant, China

## Abstract

**Background:**

Although the prevalence of malnutrition among Chinese infants has decreased, micronutrient deficiency is still common. This study aimed to describe and compare the status of micronutrient deficiency and its association with dietary variety and socio-demographic features among infants from urban and rural China.

**Methods:**

A cross-sectional study was performed on 1200 children aged 18-month-old from rural villages in Yunnan and an urban city in Shanghai. Information on food intake was obtained from a 24-h dietary recall technique. Anthropometric measures, dietary diversity score (DDS), food variety score (FVS), and mean adequacy ratio (MAR) were calculated and compared. Correlations between DDS, FVS, MAR, NAR, and anthropometric measures were examined.

**Results:**

Compared with urban area, DDS, FVS, and NAR of most micronutrients of infants from rural areas were significantly lower. These data corresponded to significant lower Z-scores of physical growth in rural infants. DDS, FVS, and NAR were positively correlated to anthropometric measures.

**Conclusion:**

Infants from rural areas consumed a significantly lower amount of micronutrient and had worse anthropometric measures. Both DDS and FVS could be used in dietary assessment studies on children. This is the first research quantified the difference in dietary diversity and micronutrient status of infants in rural and urban areas of China. Our work can potentially serve as a guide to infant feeding recommendations.

## Introduction

China has undergone rapid growth of the national economy during the past several decades. However, what lies under such development was the widening gap between urban and rural areas to such an extent that the living standard of people in many remote areas has lagged behind that of capital cities for several decades. Disparities also exist in the nutritional status of children. Although malnutrition is now less commonly caused by inadequate intake of calories and protein, the prevalence of micronutrient deficiencies has increased, owing to poor feeding during early stages of newborns(1). The prevalence of micronutrient deficiency ranges was from 3.5% to 50% on almost all micronutrients especially in rural areas such as Tibet, Jiangxi, Gansu and Yunnan Province (2). Several initiatives have been implemented by the Chinese Ministry of Health to improve child nutrition such as the “Mother and Baby Package Project” between 2006 and 2010, however, quantitative progress of such initiatives was unknown due to the lack of data primarily because accurate and sensitive clinical and biochemical indicators are difficult to obtain on a wide range of micronutrients (3). One way of collecting and analyzing the micronutrient status of children is by using the quantitative dietary records. The records utilize dietary analysis software to evaluate children’s dietary intake of various micronutrients. The process, however, is very time-consuming and difficult to implement, as it requires specialized operators and caregivers with adequate literacy in nutrition to provide accurate inputs. It is, thus, important to develop a simple and yet reliable indicator as a surrogate measure to report child nutrition especially catered to poorer areas where residents are generally unfamiliar with nutritional concepts.

Individual dietary diversity (IDD) served as a good candidate for this purpose (4). IDD calculates the dietary diversity score (DDS), which represents the frequency of a certain type of food in the past one month and the food variety score (FVS), which evaluates the overall variety of individual’s food intake over the past seven days. DDS and FVS can be used reliably to reflect micronutritional status and they are correlated to anthropometric measurements in children.

Therefore, the present study aimed to assess the nutritional status and its determinant factors of infants age 18-months in rural China and compare them with those living in urban areas using the WHO standards; and establish the suitability of DDS and FVS in predicting micronutritional status and their associated functional outcomes in Chinese population.

## Materials and Methods

### Study design and eligibility criteria

This study is part of our large study entitled “Development and Health of Rural Chinese Children Fed Meat as a Daily Complementary Food from 6 to 18 Months of Age” conducted in Yunnan Province and Shanghai. Eighteen months-old infants were recruited from rural villages in Yunnan and were compared with age-matched children from Shanghai presented for routine well-child check.

The study protocol was approved by the Ethics Committee of Xinhua Hospital affiliated to Shanghai Jiao Tong University School of Medicine. Written informed consent was obtained from the mother of each infant.

Inclusion criteria include healthy termed singleton infants, 17 to 19 month-old, with birth weight over 2500 gr, without metabolic or physical problems, were breastfed for at least 6 months, and agreed to follow the study protocol. Exclusion criteria include preterm/low birth weight infant, previous or current use of high dose supplements or medications that might affect nutrient metabolisms. The study was designed to include only apparently healthy mothers. Our initial recruitment and continuous screening process, therefore, have excluded mothers with history of preeclampsia, eclampsia, preexisting hypertension, gestational hypertension, preexisting diabetes, gestational diabetes, all thyroid disorders, infectious disease, and/or substance abuse issues. Only mothers conceived naturally were recruited.

### Data collection

#### Information from Questionnaire

Our questionnaires include background information (the main caregiver’s education, mother’s pre-pregnancy weight, height, and family income) and the infant’s feeding practices such as consumption of food groups during the week or the past month. The questionnaires were developed based on the WHO feeding recommendations for infants and related literature (5).

### Anthropometric measurements

#### Body Mass Index

All anthropometric measurements were conducted by two trained researchers. The Body Mass Index (BMI) was determined by calculating participants’ weight in kilograms over measured height in meters squared (kg/m^2^). Weight and height were measured using a digital weighing scale (Seca, Germany) and a standard clinical stadiometer (Seca, Germany). Weight was measured to the nearest 0.005 kg and height to the nearest 0.1 cm. Body weight was measured twice continuously; if the difference between the two measurements was less than 0.01 kg, the first measurement was recorded. Height was also measured twice continuously; if the difference between the two measurements was less than 0.4 cm, the mean height of the two measurements was recorded. Continuous measurements were repeated and the last measurement was recorded only when the difference between the two measurements was in the acceptable range.

#### Z-score

Z-score was calculated by using the software WHO Anthro 3.0.1 (version 3.0, April 2009). Parameters included were weight-for-age Z-score (WAZ), weight-for-height Z-score (WHZ), and length-for-age Z-score (LAZ). Underweight, stunting or wasting were defined as WAZ < −2, LAZ < −2, and WLZ < −2, respectively. Overweight was defined as WLZ > +2.

#### Nutrient intake

The information regarding infant feeding practices was obtained based on a 24-h and 7-d food recall through on-site face-to-face interviews with the parents or main caregivers by a trained clinical research assistant during each visit. DDS was calculated as the sum of all food groups consumed by a child. FVS was calculated by adding all food items consumed by the subjects during the seven days. Only food items categorized in the 8 food groups were included. The 8 food groups included in our study were carbohydrates (i.e. grains), legumes and nuts, dairy (milk other than breast milk), meat (i.e. poultry or internal organs such as liver, seafood (i.e. fish, shrimp or crabs), eggs, fruits, and vegetables. We selected these food groups based on the recommendations of WHO, which include all major nutrients important for the development of infants as well as food commonly available and consumed in the Chinese community.

#### Nutrient adequacy

Micronutrient contents in the food consumed were measured using nutrition data system for research software (NDSR, Nutrition Coordinating Center, Minnesota, USA). Nutrient adequacy ratio (NAR) was calculated for 10 micronutrients by comparing the distribution of estimated intake with the estimated requirement for that micronutrient as recommended by the WHO (6, 7). The mean adequacy ratio (MAR) was calculated as the average of all NARs (8).

#### Statistical analysis

Descriptive analyses were conducted on the socio-economic and anthropometric parameters of the study populations and their associated nutrient intakes were compared and analyzed. After adjusting for confounders, analysis of covariance was used to analyze the difference on growth in the different groups. Variables were analyzed using nonparametric analysis. Spearman Correlation Coefficients were calculated between nutrient intakes and dietary diversity scores. All tests were 2-sided with statistical significance determined at *P*<0.05. Statistical analyses were performed using SPSS (Ver. 17, IBM Corp, USA).

## Results

### Loss to follow-up

A total of 1200 infants (851 in the rural group and 349 in the urban group) were enrolled. Overall, 98 infants were excluded because of incomplete questionnaire (70 in the rural group and 28 urban).

### Sample characteristics

Both genders were equally represented in the study population with 540 boys (50.37%) and 532 girls (49.63%). All demographic and anthropometric data were given in Table 1. Infants participating had a mean age of 17.98 ± 0.22 months, mean weight of 10.28 ± 1.39 kg, and mean height of 78.74 ± 3.79 cm. The mean WLZ, WAZ, LAZ were 0.18 ± 0.91, −0.33 ± 0.11, −0.98 ± 0.31, respectively. 247 infants (23.04%) were found to have stunting (LAZ < −2), 5 (0.47%) had wasting (WLZ < −2), and 61 (5.69%) were underweight (WAZ < −2). All of these 313 infants were from the rural areas. Infants with LAZ ≥ 2, or WLZ ≥ 2, or WAZ ≥ 2 were all from the urban area (Table 1).

**Table 1:** Urban-rural differences in socio-economic and anthropometric parameters

***Parameters***	***Rural (n=751)***	***Urban (n=321)***	***P***
Family income (RMB)	7680 ± 2000	79800 ± 40000	<0.001
Highest education above high school (% of recruited subjects)	8.0 ± 1.2	65.8 ± 5.1	<0.001
Mother’s BMI ≥ 24 kg/m^2^ (% of recruited subjects)	17.0 ± 2.5	18.6 ± 3.4	> 0.05
Weight (kg)	9.68 ± 1.03	11.7 ± 1.08	<0.001
Length (cm)	77.02 ± 2.78	82.76 ± 2.64	<0.001
WLZ	−0.09 ± 0.81	0.82 ± 0.80	<0.001
WAZ	−0.81 ± 0.86	0.80 ± 0.72	<0.001
LAZ	−1.58 ± 0.96	0.41±0.88	<0.001

All weight, length, WLZ, WAZ, and LAZ are corrected for socio-economical measures. Data in mean ± SD

In regards to socio-economical status, significant differences were found in family income, main carer’s educational level, mother’s BMI. Total family income of families was below 10000 RMB (7680 ± 2000) in the rural areas while families in urban area all earned between 60000 to 150000 RMB (79800 ± 40000).

The education level of main caregivers in rural was mostly below high school level (56% below junior) compared with those in urban mostly tertiary educated (75% above senior high school). Lower rate of overweight/obesity (17%, BMI ≥ 24 kg/m^2^) was found in rural mothers compared with urban mothers (18.6%, BMI ≥ 24 kg/m^2^).

### Urban-rural differences in anthropometric measures

At 18 months of age, rural infants clearly lacked behind in their developmental parameters as measured by WLZ, WAZ, and LAZ. The differences were still significant even after correcting for socio-economic data.

### Nutrient intake for all infants

When all infants were considered as a whole, total energy intake and total protein intake were found to be 864.45 ± 79.14 kcal/day (NAR of 91.17%) and 2.36 ± 0.7 g/day (NAR of 229.12%) respectively. All micronutrients, except vitamin A and calcium, were above WHO recommended level indicated by NAR>100% (Table 2).

**Table 2:** Micronutrient intakes of all infants as compared with WHO recommended levels

***Micronutrients***	***Intake***	***WHO recommendations***	***NAR (%)***
Vitamin A (μg/d)^$^	429.8 (170.5–442)^@^	400	66.0 (42.7–110.5)
Vitamin C (mg/d)	32.8 (12.9–58.3)	30	109.3 (43.1–194.2)
Vitamin B1 (mg/d)	1.15 (0.9–1.5)	0.5	229.9 (178.5–295.8)
Vitamin B2 (mg/d)	0.7 (0.4–1.0)	0.5	146.9 (86.8–207.0)
Niacin (mgNE/d)^*^	10.3 (8.2–12.7)	6	171.4 (136.1–211.8)
Vitamin B6 (mg/d)	0.85 (0.67–1.07)	0.5	170.4 (133.0–213.7)
Vitamin B12 (μg/d)	1.2 (0.5–2.1)	0.9	138.2 (57.8–228.9)
Calcium (mg/d)	340.5 (186.0–436.7)	500	64.1 (37.2–87.3)
Iron (mg/d)	8.6 (6.8–10.5)	4.8	178.1 (141.8–218.5)
Zinc (mg/d)	4.9 (3.9–5.9)	4.1	119.5 (94.7–144.3)

$ excludes beta-carotine.

@ Median (P25–P75).

* niacin equivalent

### Urban-rural differences in micronutrients intake

Adopting the recommendation from the WHO, infants from both urban and rural areas were deficient in vitamin A and calcium intakes but rural infants were also deficient in vitamin C and B12 (Table 3). In comparison between urban and rural infants, a significant difference was found in all micronutrients intake except for vitamin A and niacin, although those not mentioned above met the target of the WHO. MAR of urban infants was significantly higher than that of rural infants.

**Table 3:** Urban-rural differences in micronutrient sufficiency

***Micronutrients NAR (%)***	***Rural (n=751)***	***Urban (n=321)***	***Z***	***P***
Vitamin A	65.5 (33.0–134.9)	66.8 (58.0–79.6)	−0.851	0.395
Vitamin C	72.3 (29.9–154.4)	160.3 (129.1–270.1)	−13.619	<0.001
Vitamin B1	223.0 (169.8–288.2)	246.3 (198.3–301.5)	−3.499	<0.001
Vitamin B2	110.0 (73.2–166.4)	207.0 (183.2–241.0)	−16.984	<0.001
Niacin	169.3 (129.9–222.1)	173.9 (144.9–200.6)	−0.820	0.412
Vitamin B_6_	159.4 (121.0–208.4)	185.7 (159.0–234.0)	−7.449	<0.001
Vitamin B_12_	82.6 (40.8–163.1)	233.9 (188.2–295.6)	−18.944	<0.001
Calcium	51.0 (31.2–81.0)	81.6 (68.8–93.1)	−11.911	<0.001
Iron	165.5 (129.0–214.1)	198.7 (173.6–222.2)	−6.923	<0.001
Zinc	115.7 (88.7–146.3)	125.6 (108.1–143.0)	−3.285	0.001
MAR	154.6 (120.7–201.7)	194.5 (172.9–229.5)	−10.198	<0.001

NAR > 100 indicates the WHO recommendations met or exceeded.

Urban and rural infants scored a DDS of 9 and 14 respectively. Similarly, urban infants also scored higher in FVS compared with rural infants (6 vs. 8). Only 4 out of 751 rural infants had a full FVS of 8 whereas 271 of 321 urban infants scored 8 in FVS. Taken together (DDS + FVS), urban infants clearly had a much high dietary variety compared with rural infants (Table 4).

**Table 4:** Differences in dietary variety scores of urban and rural infants

***Dietary variety***	***Rural (n=751)***	***Urban (n=321)***	***Z***	***P***
DDS	9 (8–10)	14 (13–15)	−25.326	<0.001
FVS	6 (5–6)	8 (8–8)	−25.305	<0.001
DDS+FVS	15 (13–17)	22 (21–23)	−25.341	<0.001

### Associations between dietary diversity, nutrient adequacy and anthropometric measures

We found a significant association between NAR and DDS and FVS individually and when taken together (DDS + FVS) in all micronutrients except for niacin. All NAR, DDS or FVS were also positively associated with all developmental parameters (Table 5).

**Table 5:** Associations between NAR, DDS, FVS and developmental parameters

***NAR (%)***	***DDS***		***FVS***		***DDS+FVS***		***WLZ***		***WAZ***		***LAZ***	
	***r***	***P***	***r***	***P***	***r***	***P***	***r***	***P***	***r***	***P***	***r***	***P***
Vitamin A	0.106	0.001	0.068	0.026	0.005	0.875	0.022	0.047	0.038	0.219	0.095	0.002
Vitamin C	0.489	<0.001	0.436	<0.001	0.236	<0.001	0.346	<0.001	0.355	<0.001	0.474	<0.001
Vitamin B1	0.158	<0.001	0.115	<0.001	0.083	0.006	0.117	<0.001	0.113	<0.001	0.146	<0.001
Vitamin B2	0.566	<0.001	0.519	<0.001	0.251	<0.001	0.404	<0.001	0.439	<0.001	0.556	<0.001
Niacin	0.084	0.006	0.038	0.214	0.049	0.110	0.063	0.038	0.059	0.054	0.071	0.020
Vitamin B_6_	0.303	<0.001	0.236	<0.001	0.151	<0.001	0.221	<0.001	0.227	<0.001	0.071	0.020
Vitamin B_12_	0.584	<0.001	0.553	<0.001	0.264	<0.001	0.434	<0.001	0.475	<0.001	0.578	<0.001
Calcium	0.425	<0.001	0.387	<0.001	0.183	<0.001	0.301	<0.001	0.332	<0.001	0.418	<0.001
Iron	0.209	<0.001	0.183	<0.001	0.132	<0.001	0.171	<0.001	0.149	<0.001	0.202	<0.001
Zinc	0.217	<0.001	0.170	<0.001	0.088	0.004	0.126	<0.001	0.129	<0.001	0.205	<0.001
MAR	0.379	<0.001	0.322	<0.001	0.186	<0.001	0.270	<0.001	0.272	<0.001	0.364	<0.001

## Discussion

Our study analyzed dietary diversity measured in DDS and FVS and their correlations with NAR of all micronutrients as defined by the WHO recommended amount and anthropometric measurements of 18 month-old children in rural and urban areas of China. Micronutrient intake for Chinese infants was largely adequate but such conclusion can be misleading, as significant differences were found between infants living in rural villages and those in urban cities of China. In the population of rural children we studied, the intake of vitamin A, vitamin C, vitamin B12, and calcium were significantly lower than the recommended level. Moreover, both DDS and FVS correlated well with NAR and could reliably be used to estimate MAR of micronutrients in children.

Globally, micronutrient deficiencies remain a concern in the public (9). Micronutrients, though only a small quantity is required, have a significant and long-term impact on children’s growth and development. Vitamin A deficiency was a nutritional problem in children in China, particularly those living in the poor western area, having a mother of minority ethnicity or with poor education (10, 11). Unfortunately, despite strategy of vitamin A supplementation has since been put into the National Programme of Action for Child Health in China (2001–2010), vitamin A deficiency is still present and our data showed that it is likely to be due to persistent low intake. Other reasons include a short duration of breastfeeding among children with low socioeconomic status, also contribute to low serum retinol concentration (10, 11). From a public health perspective, children living in farming counties such as Tibet had higher serum vitamin A, suggesting a diet rich in milk and meat products both for the children and their mother is effective in preventing vitamin A deficiency (12). In addition to promoting vitamin-A-rich foods, vitamin A supplementation should be offered during public vaccination consultation. Nutrition education and fortified complementary feeding should also be promoted to mothers.

In regards to vitamin C, Chinese typically lack ascorbic acid in their diet. The problem is made worse in low-income households as fresh fruits and vegetables are relatively expensive. Unfortunately, most fruits are also major contributors of a number of nutrients including vitamins A and C, folate, and potassium, all of under-consumed in children from low-income family. The same goes with vitamin B12. The best sources of vitamin B12 include eggs, milk, cheese, milk products, meat, fish, shellfish, and poultry. Regular consumption of these foods also incurs significant costs and may not be affordable for rural families. Our result showed that vitamin B12 intake was too low, consistent with two previous surveys conducted in Yunnan and Chongqing in 2006, which reported vitamin B12 deficiency in 4.5% and 10.7% of children in Yunnan and Chongqing, respectively (13, 14). The above mentioned foods are also rich in iron. Although we did not find any deficiency in iron intake, rural children are nonetheless at greater risk of iron deficiency compared with urban children.

We identified that one of the major contributors to micronutrient deficiency is the lack of dietary diversity. We found rural children had less dietary diversity overall, especially among vegetable, fruit, and animal food groups, leading to a greater stunting rate and poorer physical and possibly mental development. Childhood stunting is strongly associated with shorter height, less education, reduced economic productivity, increased risk of death and disability-adjusted life-years in adulthood (1, 15, 16). The lack of dietary diversity can be attributed to both cultural and structural factors. Culturally, Chinese diets are still based on plant foods; the energy density of these diets depends heavily on cereals. This is especially true for rural children whose diet is rather monotonous and consists primarily of high glycaemic index carbohydrate such as flour or rice. WHO recommends that the diet of 18-month-old children should consist of foods from at least five to six categories or ideally from all eight categories of complementary foods (grains, vegetables, fruits, fish, meat, eggs, beans and milk) (6, 17–21). Correspondingly, DDS should then be at least 12 and/or FVS should be ideally over 8. Our data showed that rural children are yet to meet such target. The structural causes include lack of nutrition education, poor access to nutritionists, limited resources, or even parents who might be deficient in these micronutrients themselves. Clearly, demographic factors play a role. Rural families are typically poorer and have less education than their urban counterparts; both factors have been shown to be positively correlated with the direction and significance of rural-urban difference in dietary quality.

The strengths of our study include the large sample size, which facilitated the comparison between the two groups of children despite that we only selected children of a very narrow age range. We were also able to obtain detailed information in dietary recall. An additional strength is the availability of biological data for this large sample of participants. As we used the NDSR in this study, participants only need to describe the size of food rather than measuring the actual number in grams. This helped to ensure accurate recording as describing food rather than quantification was technically less demanding to parents especially rural parents with considerably lower education. After inputting the cooking method, such as deep fried or stirred fried, the usage of oil could be automatically calculated. We found that the NDSR was able to capture all Chinese dietary practices and food consumed. The NDSR also includes a module for dietary supplements evaluation to ensure that the nutritional intake and supplement sources could be captured and quantified.

One primary limitation of the present study lies in its cross-sectional design that disallows a sequence of temporality to be established. Future prospective investigations are warranted to clarify the findings herein. Another limitation is that we had no documentation on mothers’ prenatal dietary practice or breast milk compositions. Our study focused on micronutrient deficiency in dietary practice and that may not translate to the actual deficiency, only diagnosed by blood tests. We infer that if these children were to continue on such dietary practice for a longer period of time, they would inevitably be at risk of the actual deficiency. Several other potential confounders were not adjusted in the logistic regression such as infections, safety of water or food. These factors could potentially affect the accuracy of our findings.

## Conclusion

Overall, the average micronutrient intakes were significantly lower among rural children in China compared with urban children. Several micronutrients such as vitamin A, C, B12, and calcium were below the WHO recommended intake. In addition to structural factors, such as poor socio-economic status, access to a variety of foods, and poor education, cultural factors such as the Chinese diet and feeding habits should not be overlooked. Several national initiatives that have been put in place to educate and provide supplementations to rural children did not seem to have abolished the problem. Significant investments in public health will be required to encourage public engagement and compliance. DDS and/or FVS were positively correlated to NAR and developmental parameters. We recommend health policies to be targeted in increasing dietary diversity and fostering nutrition education to help reduce the prevalence of micronutrient deficiencies among Chinese children.

## Ethical considerations

Ethical issues (Including plagiarism, informed consent, misconduct, data fabrication and/or falsification, double publication and/or submission, redundancy, etc.) have been completely observed by the authors.
